# Vaidya V. B. Mhaiskar

**DOI:** 10.4103/0975-9476.72616

**Published:** 2010

**Authors:** Vaidya Narendra M. Pendse

**Affiliations:** *Ayurvedic Practitioner, Pune*

**Figure:**
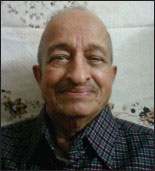
Vaidya V. B. Mhaiskar

Vaidya Vinayak Bhaskar Mhaiskar was born on March 18,1929 in the small village of Bhose near Sangli in Maharashtra state, and received his primary education in the nearby village school. When he was about to complete his matriculation examination in Sangli, his father passed away. At that juncture, due to the social upheaval taking place in the aftermath of Mahatma Gandhi’s death, he left home for higher education in Pune.

Having a singular desire to learn Ayurveda, he gained admission to the reputed Ayurveda Mahavidyalaya, Pune (later Tilak Ayurveda Mahavidyalaya). There, he studied Sanskrit for 2 years under the learned scholar Shende Shastri. The greatest influence on him, however, were the exemplary teaching and clinical skills of Vaidya B. V. Gokhale (alias Mama), who fascinated him, and remained a continued inspiration for the rest of his life. He graduated D.A.S.F. in 1952. In 1958, he completed his A.V.P., Ayurvidya Parangat, in Kayachikitsa from Tilak Maharashtra Vidyapeeth. Soon after, on the recommendation of his mentor Gokhale, he joined the post graduate training center of Ayurveda (PGTCA), Jamnagar, as a resident medical officer, working there for 13 years until 1970 in various capacities primarily in the Department of Kayachikitsa. After a short period at Bhavnagar, he joined the Ayurveda College in Vadodara, Gujarat, in 1971, where he rose to be Principal, retiring in 1987.

His students deeply respected his exemplary bedside teaching of clinical skills. Like Gokhale, his mentor he emphasized the importance of making an Ayurvedic diagnosis based on the classical Ayurvedic texts, and of providing precise Ayurvedic treatment. It was, and still is, his norm to quote verbatim from the classics of Charaka and Sushruta, when enunciating a particular diagnosis or treatment. Although an acknowledged expert in Ayurveda, he is also very well read in Western Medicine, and aware of the principles of Chinese medicine, acupuncture, and chiropractic. He is a master of both Shodhana (deep tissue cleansing / detoxification / rejuvenative techniques of Panchakarma) and Shamana (primarily palliative procedures) modalities of treatment.

As author of more than 200 papers, he is respected for the consistent quality of his writing in various Ayurvedic journals in Gujarati, Hindi, Marathi, and English, and his expert presentations in seminars and conferences. He published case series on various pathologies: ‘Jwara’ (akin to various types of hyperthermia), ‘Kushtha’ (various skin disorders), ‘Vata-vyadhis’ (various primarily neurological but multisystemic energy impairments), ‘Hridroga’ (cardiac disorders) (*Management of Hridroga*, CCRAS publication Vol.1, No.1, pg. 25-34), ‘Amavata and Sandhigat Vata Vyadhi’ (respectively akin to rheumatoid arthritis and osteo-arthritis), ‘Switra’ (vitiligo), ‘Vrischika-dansha’ (scorpion sting), ‘Shiro-roga’ (various types of headache), and ‘Marma-abhighataja vyadhis’ (diseases arising from injury to the ‘Marmas’ – vital points in the body).

Some topics on which he wrote extensively have rarely been touched by others. Each article’s clinical aspects have rich inputs from underlying, conceptual, ‘Shastriya’ principles. For example, he made studies in epidemiology, a comparatively untouched area of Ayurvedic practice, writing articles on the prevalence of diseases and formulations used. The first was based on 23,082 available OPD case papers at PGTCA, Jamnagar, in the year 1963. Only 4,389 cases had clear diagnosis given. That paper analyses the incidence of 87 diseases and the 343 formulations most commonly used for them (in English pg. 1-18, Nagarjun, July 1966 and in Hindi pg. 33-39, Ayurvedalok, January 1966 and pg. 98-100, Ayurvedalok, April, 1966) Another research article was based on 12,816 OPD case papers at the Ayurveda College, Vadodara, in the year 1971-72 (AYU, 1973, April, 9-20.

In 2002, on suggestion of Dr. P.N.V. Kurup, Mhaiskar’s series of articles in ‘AYU’ on Srotas, was published by GAU in Hindi as the book *‘Srotovidnyan’*. It is still one of the best resource books on the topic, especially regarding ‘swa-gunas’ (normal properties) of the various *srotas* in the body. Another book published by GAU was also the outcome of various articles: ‘Atyayik Chikitsa’, emergency care in Ayurveda, elaborates the subject’s various conceptual and practical aspects.

A hallmark of his writings is a commendable ability to collate various references from the available ‘Tikas’ (commentaries on various Ayurvedic classics) and so draw a complete picture for the reader. One of his favorite commentators is Dalhanacharya - the scholar commentator on the classic ‘Sushruta’ samhita. An outcome of his command over the subject was an article on ‘Dalhanacharya’s Nibandhasangraha commentary’, which appeared in the commemorative volume on the late Vd. Purushottamshastri Nanal (1993, Madhavi Prakashan, Mumbai). Many commentators refer to ‘iti loke’ (literally, - as is said by the people, i.e., locally). In order to illustrate the fact that Dalhanacharya had probably traveled a great deal throughout the Indian subcontinent, and must also have been conversant with India’s various languages and botanical, geographical, and cultural diversity, Mhaiskar leads the reader through Dalhanacharya’s 313 references to ‘iti loke, and their nuances. Chakrapanidutt’*s commentary* on ‘Charaka’ Samhita only uses ‘iti loke’ 30 times; Arundutt commenting on ‘Ashtanga-Hridaya’ only mentions it 26 times.

Some of his articles concerned the concept of ‘Vaidya-sthanam’, a place primarily inhabited by Vaidyas or famous for its Vaidyas. For example, Dalhanacharya refers to a place called Ankola, near Mathura in North India as his place of origin. Mhaiskar researched the name extensively and reported a hitherto unidentified Vaidyasthana named Ankola near Hubli in northern Karnataka in South India. At the time of reporting, the town still had six practicing Vaidya families, each of them specialists in the traditional treatment of specific diseases including, ‘Pakshaghat’ (akin to paralysis), ‘AndaVruddhi’ (akin to hydrocele), ’Bhagna’ (akin to bone fractures), ‘Apasmara’ (akin to epilepsy), ‘Kamala’ (akin to Jaundice), etc. With the help of local Vaidyas, especially V. N. Dattatreya, he painstakingly brought out this forgotten information. (pg. 8-10, Madhujeevan, April, 1999).

He followed this article up with a report of another Vaidyasthana named Pacchegaon or Paschimgram, 50 miles from Bhavnagar in Saurashtra in western India, which has been a Vaidyasthana for at least 800 years. Many families of ‘Prashnora Brahmins’ who live there traditionally practiced Ayurveda, Jyotish astrology, read the Bhaghavatam (a religious text), and practiced farming. A famous member of the ‘Prashnora-community of Vaidyas was the renowned ’Rasavaidya’ (expert in preparation of medicines from mercury and other metals) Zandubhattji – founder of the ‘Zandu’company. The article recounts several examples of remarkable Ayurvedic diagnostic and treatment skills manifest in the lives of many of the Vaidyas who originated there. Many of the families were expert in preparing particular medicines such as Bhasma (calyx), Kwatha (herbal decoction), or specialized in treating particular classes of pathology such as Madhumeha (akin to diabetes mellitus), Pakshaghat (akin to paralysis), or Grahani (akin to various gastrointestinal afflictions). A peculiarity of this group of Pacchegaon Vaidyas was their habit of not asking patients for money. Patients would give cash or in kind according to their inclination. Keeping only what was genuinely needed, the Vaidya would donate the rest to the needy. (pg. 4-7, Madhujeevan, 2000).

In 2006, together with Dr. S.Y. Wakankar (Dep. Director, Oriental Institute, M.S. University, Vadodara), Mhaiskar edited a very rare, previously unpublished manuscript of Maharishi Palakapya’s ‘Gajashastra’, (Folio 393, Acc no. 1853) including the Sanskrit commentary by Bhavasandarshini of Anantakrishnabhattaraka. It was published as a 462 page book with English translation (2006, Bharatiya Kala Prakashan, 3421-A, Narang Colony, Trinagar, Delhi-110035). It differs from Hastayurveda, Pune edition, and Gajashastram, Tanjore edition, as these deal solely with Ayurveda and elephants, while Mhaiskar’s includes general information e.g. life-span, without giving Ayurveda undue importance. Mhaiskar analyzed Sushruta’s inputs quoting Dalhanacharya’s Nibandhasangraha commentary and its observations on types and sub-types of land and water dwelling animals and birds, including peculiarities of their shape, structure and behavior - a Vedic zoology.

Recently Mhaiskar published a book on ’Nadi-vigyana’ (pulse diagnosis) called ‘Nadi-vivechana’. (2009, Madhavi Prakashan, Mumbai). This 93 page book in Marathi provides an extensive database from all the popular, available original texts of ‘Nadi-vigyana’ and shares his life-long experiences of using it as a means of assessment.

Presently he is working on an English textbook on Vatavyadhi. He has already compiled more than 300 pages of text and references. The book comprises extensive cross-referencing from the classics and commentaries besides his rich clinical experiences. Students, teachers and scholars of Ayurveda will certainly benefit once it is published.

Mhaiskar is a respected national figure in Ayurveda, who advises universities, Government Institutions and NGO’s. He has been associated as a mentor / consultant with unique projects including Triskandha Kosha (the three volume encyclopedic reference work (J-AIM ref. Gadgil, Issue 1) of the Tilak Maharashtra Vidyapeeth, Pune, C-DAC’s Ayusoft and Madhavnidana software, and the subsequent Pulse project. He is a recognized Guru, under Rashtriya Ayurveda Vidyapeeth’s ‘Gurukul’ program, a prestigious central government initiative, and has traveled extensively in India, the USA and elsewhere abroad in service of Ayurveda.

Mhaiskar has won many awards: the Silver medal from the Ayurveda Academy, Vijayawada in 1976; the Life time Achievement Award by the Rashtriya Shikshan Mandal, Pune, in 2002; and the Maharshi Annasaheb Patwardhan Puraskar by the Vaidya Khadiwale Vaidyak Sanshodhan Sanstha, Pune, in December, 2008.

Vaidya Mhaiskar is known for his helpful nature. Students from all over India seek his guidance, and he is always very supportive, quoting easily from classics and commentaries, and sharing his clinical experiences. His knowledge is encyclopedic and he still keenly follows positive developments in the field of Ayurveda in India and abroad. His sharp memory enables him to quote verbatim from books on the history of India and both World Wars, and from the great personae of his era. He is particularly appreciative of the life and contributions of B.V. Gokhale, Maharishi Mahesh Yogi, P. M. Mehta, C. Dwarkanath, Vasudevbhai Dwivedi, V. J. Thakar, C.P. Shukla, Bhaskarbhai Hardikar, K. C. Barot, K. Sadashiv Sharma, H. S. Kasture, P. N. V. Kurup, B. P. Nanal, Y. G. Joshi, and M. V. Kolhatkar.

Mhaiskar’s family includes his wife, Mrs. Vimaltai Mhaiskar, an accomplished Ayurvedic gynecologist and obstetrician, and their three children, with whom he lives in Vadodara.

